# Automated inline extracellular volume (ECV) mapping

**DOI:** 10.1186/1532-429X-17-S1-W6

**Published:** 2015-02-03

**Authors:** Bruce S Spottiswoode, Martin Ugander, Peter Kellman

**Affiliations:** 1 Siemens Healthcare, Chicago, IL, USA; 2 Department of Clinical Physiology, Karolinska Institute and Karolinska University Hospital, Stockholm, Sweden; 3 National Heart, Lung, and Blood Institute, National Institutes of Health, Bethesda, MD, USA

## Background

Quantitative extracellular volume (ECV) estimates based on T1 mapping have potential for characterizing cardiomyopathies with diffuse fibrosis and edema [1,2]. An unsupervised approach for generating pixel-wise ECV maps was recently introduced [3]. In this work we extend the algorithms to include saturation recovery-based T1 mapping and present an inline implementation of these methods, offering a fast and fully automated approach for generating pixel-wise ECV maps as Dicom images on an MR scanner console.

## Methods

**Figure 1:**
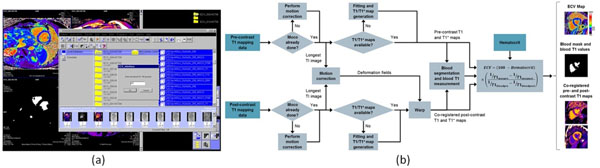
(a) User interface for ECV mapping on the scanner console. The entire patient or a subset of images can be selected in the patient browser. The generated ECV maps, blood masks and co-registered T1 maps are saved as Dicom images in the database. (b) Core processing pipeline performed for each matching pair or pre- and post-contrast T1 mapping data.

Figure 1a shows the interface which has been implemented as an investigational prototype on the scanner host (MAGNETOM syngo MR D13 software line, Siemens AG, Erlangen, Germany). The process is launched from the patient browser, where the user can conveniently select the entire study or individual images for processing, and also enter the hematocrit. The software first identifies T1 mapping data then pairs up appropriate pre- and post-contrast datasets for each slice location. The algorithm shown in Figure 1b is then performed for each pair.

The processing path will differ depending on which source images are found, and is prioritized to minimize computation. If pre-existing T1 maps are found then these are deformed directly instead of redoing the fitting. In all cases, images with the longest inversion times from pre- and post-contrast data are spatially aligned using elastic image registration [4], and the resulting deformation fields are applied to all corresponding post-contrast data. Blood T1 measurements are made using a mask based on blood T1 thresholds, morphological image processing, and a histogram classification to remove outliers.

Preliminary validation was performed on data from 20 subjects. The scans contained multiple slices of T1 mapping data, with a mixture of MOLLI, ShMOLLI and SASHA acquisitions, and a total of 112 matching pairs of pre- and post-contrast data. The processing time per slice was recorded and the effect of motion correction was assessed visually relative to manually drawn landmarks on the T1 and ECV maps.

## Results

**Figure 2:**
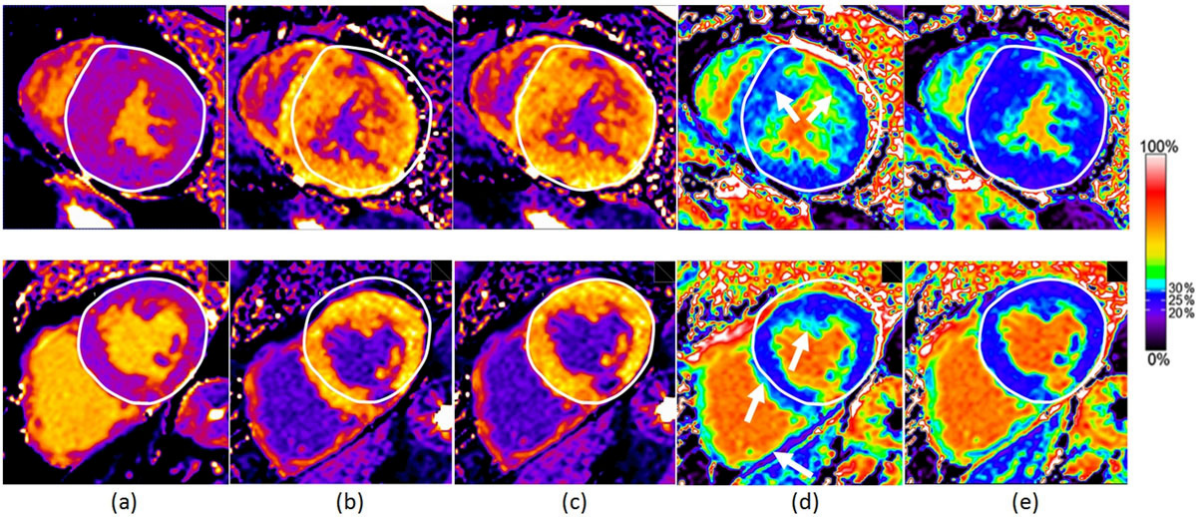
Effect of motion correction for pixel-wise ECV mapping on a subject with hypertrophic cardiomyopathy (top row) and chronic myocardial infarction in the inferior LV wall (bottom row). (a) pre-contrast T1 map after motion correction; (d) ECV map using maps (a) and (b); (e) ECV map using maps (a) and (c). The white epicardial reference contour was manually drawn on the pre-contrast T1 maps, and the white arrows in (d) illustrate some areas with missing myocardium or artificially elevated ECV due to breath hold positions not being spatially aligned. The colormap proposed in [3] has been used, where deep blue represents a normal ECV range of 20-30%.

ECV maps were successfully generated for all 112 cases. The processing time for the pipeline in Figure 1b with pre-existing T1 maps was 0.7 seconds per slice. For large studies (~2500 images), image loading and sorting can take up to 10 seconds. In 3 cases at short axis apical slice positions the algorithm failed to produce a reliable blood mask because of insufficient ventricular blood pool area. Figure 2 shows the benefits of motion correction for two subjects.

## Conclusions

A user friendly software tool for generating pixel-wise ECV maps as Dicom images on an MR scanner has been developed. The algorithms are fully automated and include a variety of processing options for both inversion and saturation recovery-based T1 mapping data.

## Funding

N/A.
